# MarkerSet: a marker selection tool based on markers location and informativity in experimental designs

**DOI:** 10.1186/1756-0500-1-9

**Published:** 2008-03-26

**Authors:** Olivier Demeure, Frédéric Lecerf

**Affiliations:** 1 INRA, UMR 598 Génétique Animale, F-35000 Rennes, France; 2 Agrocampus Rennes, UMR 598 Génétique Animale, F-35000 Rennes, France; 3 IFR 140, GFAS, F-35000 Rennes, France

## Abstract

**Background:**

The recent sequencing of full genomes has led to the availability of many SNP markers which are very useful for the mapping of complex traits. In livestock production, there are still no commercial arrays and many studies use home-made sets of SNPs. Thus, the current methodologies for SNP genotyping are still expensive and it is a crucial step to select the SNPs to use. Indeed, the main factors affecting the power of the linkage analyses are the density of the genetic map and the heterozygosity of markers in tested animal parents.

**Findings:**

This is why we have developed a PERL program selecting a defined number of markers based on their locations on the genome and their informativity in specific experimental designs. As an option, different experimental designs can be combined in order to select the best possible common marker set. The program has been tested using different conditions of marker informativity and density with both real and simulated datasets. The results show the efficiency of our program to select the most informative markers even if there is a wide range of informativity for whole genome scan mapping analyses. In case of combination of different experimental crosses, the multidesign mode can optimize the SNP markers selection.

**Conclusion:**

Written in PERL, it assures a maximum portability to other operating systems (OS) and the source code availability for user modifications. Except for the simulation mode which could be time consuming, MarkerSet can compute results in a very short time.

## Findings

The recent sequencing of full genomes has led to the availability of many SNP markers ([1] for Human and [2] for Chicken). The current methodologies for home-made SNP sets genotyping are still expensive, meaning that only few thousands of SNPs can be used. It is then a crucial step for a specific study to select the best suited SNPs. For linkage analyses, the main criteria to increase the analysis power are the distances between markers and the ability to follow the marker's allele segregation in the experimental design. It means that the markers must be as much as possible heterozygous for phenotyped animal parents. This is why the heterozygosity in phenotyped animal parents (further called reference animals) must be included in the marker selection. In this manuscript, this heterozygosity for reference animals will be referred as informativity of the markers. From our point of view, if there are no available SNP arrays, the best strategy is a two step genotyping, with a test of a large panel of SNPs informativity on reference animals from the studied experimental design, followed by a genotyping of all the animals for markers selected based on the results of the first step. The marker selection is complicated by the fact that markers the most heterozygous in reference animals are not homogenously spaced across the genome, and the number of markers to handle has greatly increased. It is therefore not possible anymore to select the markers without dedicated software. Different tools have already been proposed to select Tag SNPs [3-11], but most of them are based on very high marker density and linkage disequilibrium information and cannot be used in exotic species and species without SNP arrays for which linkage disequilibrium information is not always available. We propose here a tool to select the best possible markers for further linkage analysis, without any use of linkage disequilibrium information. Its originality is the use of both marker location in the genome and heterozygosity in parental animals.

The MarkerSet software was written in the PERL programming language and can be downloaded with manual and example files at .

The software is designed to use already available information about markers informativity, expressed in number of heterozygous animals out of all the reference animals tested in the experimental design. This allows the use of any kind of markers, as well as their combinations if needed. If more than one experimental design is to be genotyped, a specific set of SNPs can be selected, or the marker informativity for all these experimental designs can be used simultaneously to select common sets of markers. In case of a marker set selection common to all designs, both general informativity score and experimental design specific scores are detailed, so it is possible to evaluate specifically the marker set informativity for each experimental design.

In most species, the only available information for the markers will be their physical location (especially true for SNP markers), as all the markers have not been tested on a reference population to estimate genetic distances. Nevertheless, for a QTL mapping, the genetic distances are the key points as, depending on the species, the recombination rate can highly vary. So MarkerSet uses physical distances as input and converts them into cM. This conversion can be adapted to fit the specificity of the studied species (as an example, in pigs, we can considerer that 1 cM corresponds to approximately 1 Mb).

**Figure 1:**
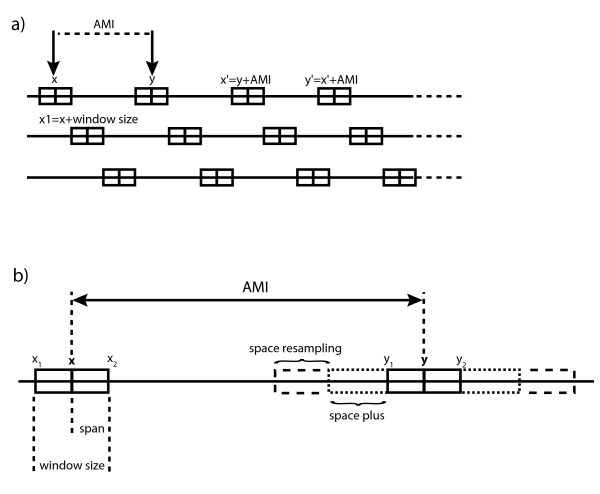
Principles of MarkerSet and main parameters. a) MarkerSet selects markers in two windows separated by the Average Marker Interval (AMI), which is the whole genome size divided by the number of markers to select. The window size is a percentage of the AMI (20% by default). Shifting iteratively the windows by the AMI gives a full genome coverage. Different sets are created by using all the possible starting points (x and y). b) Several parameters and options are available in order to improve the sets quality. The space_plus and space_resampling parameters are used to enlarge the window size in case of low (or no) informativity: space_plus is set by default as 50% of the window size on each side. This is automatically performed if the informativity of markers available is lower than the defined informativity threshold. Space_resampling is used to iteratively enlarge window size (by default +1 cM on each side at each step) until markers with informativity higher than the defined resampling threshold are found (resampling option mode).

Basically, the algorithm will select the most informative markers in two windows separated by a constant gap, and sliding on the genome (see Figure 1A). In case of a similar informativity between several markers in a window, MarkerSet will select the closest marker from the middle of the window. Using this strategy, the distance between two markers is the first criterion of selection, and the informativity is used for discriminating closely located markers. The two main variables are the first window starting point on the genome and the size of the gap separating the two windows. Depending on the number of markers to select and the size of the genome, MarkerSet will compute different window starting points to get the best genome coverage.

The gap size and the window size are defined by the average marker interval (AMI), corresponding to a ratio of the whole genome size and the number of the markers to select. The AMI percentage used to calculate the window size is defined in the config.pm file (set by default as 20% of the AMI). So, the setting of the selection window size is automatically handled by the software.

These two parameters (AMI and window size) permit to compute the number of possible starting points (i.e. the number of selected marker panels). Thus, for each combination of these parameters, a marker selection will be performed with a fixed starting point and multiple iterations over the genome (Selection Frame). At each iteration step, the starting point of each pickup box will be increased by AMI+window size (see Figure 1A).

**Figure 2:**
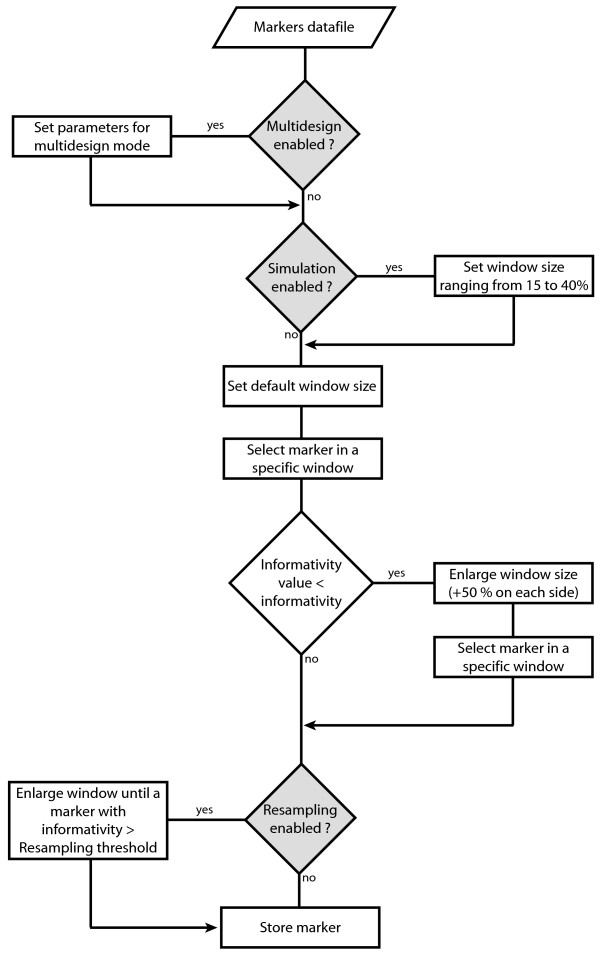
Working principle of MarkerSet.

For all analyses, an informativity threshold is set, so if the best available marker in one window has an informativity strictly lower than this threshold, the window is enlarged (space plus: 50% of the window size is added to each side of the window, as default – see Figure 1B) and a more informative marker is searched. By default, this threshold is set as half of the best possible informativity score for one marker (i.e. half of the total animals tested). If there is no marker with a higher informativity, the best previous marker is conserved, as it results in shorter distance between markers. As an option, the window size can be enlarged as long as a marker more informative than the resampling threshold (set by the user) is not found (resampling option). The window size enlargement is defined by the user through the space resampling parameter (see Figure 1B). The working principle of the software is exposed in figure 2.

**Figure 3:**
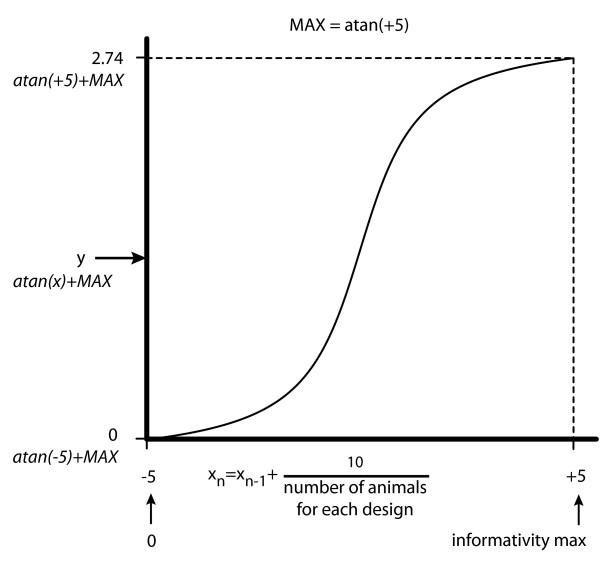
Computation of informativity weight. Empirically, this sigmoid scale is obtained by computing values between -5 and +5 with the arctangent function (corresponding to -1.37 to +1.37 transformed informativity scores). For each experimental design, we re-assign the different informativity values to a -5 to +5 scale (see Figure 3). Let X = {X_0_, X_1_, ..., X_n_} denoting the informativity status value, with n denoting the number of tested animals for one experimental design. Each informativity value is determined as X_i _= X_i-1 _+ 10/n, with X_0 _= -5 to fit a scale from -5 to +5. The informativity score values are then expressed from -1.37 to +1.37 (corresponding to -5 and +5 arctangent values respectively). The scores obtained are finally adjusted to a 0 to 2.74 range in order to get only positive score values. vertical axis represents the informativity weight, horizontal axis the informativity values.

In order to score the different obtained panel, one approach is to sum basically the informativity value for each selected marker (i.e. the number of heterozygous parental animals in our case). This approach of linear scoring is effective for markers with an extreme informativity value (i.e. 0 or 1 heterozygous animals or, on the other hand, all animals heterozygous), but it is not enough discriminative for "middle-range" marker. As an example, on a total of 6 tested animals, we prefer to give much more weight to a marker with 4 heterozygous animals than one with 3 heterozygous animals. In order to best represent the informativity of a marker, we decided to transform the informativity value of each marker on a sigmoid scale (see Figure 3). Obviously, this approach maximises or minimises the score for maximum or minimum informative markers respectively, but more importantly, discriminates "middle-range" informative markers. Finally, a panel score is obtained by summing the score values of all markers selected for this panel. In addition, the software computes some informations to describe each experimental design: maximum informativity score (i.e. the sum of informativity scores of all available markers), and the distribution of the number of markers in each informativity value class. These data are available to user in a log file.

When studying several experimental designs in the same species, user may want to compare what is the best option: to select markers perfectly fitted for each experimental design (for example heterozygous for all F1 sires), or to try to select a larger set of markers common to all experimental designs (in this case, some markers will be homozygous in some families resulting in a loss of power in the linkage analysis). To help with this dilemma, a multidesign option has been implemented. The principle of marker selection and panel scoring is absolutely the same except that the software use a global informativity value generated by summing, for each marker, the informativity value of each experimental design. Based on this global informativity, MarkerSet will select the best informative markers for the multidesign, and score it with the multidesign informativity values (score A). As mentioned above, this multidesign option should permit to evaluate which solution best fits for a number of defined markers: a set of common marker for all experimental designs or several sets of markers specific for each design. In order to measure the loss of informativity, MarkerSet will perform a simulation of marker selection specific for each design using the same selection frame (with the number of marker to select in multidesign option) and score it (score B). A ratio between multidesign score (score A) and experimental design specific score (score B) is calculated (called MD/Sim, r in the logfile). This ratio gives an estimation of the "conserved" informativity score between multidesign and design-specific marker selection: as an example, a ratio of 0.82 means that only 18% of informativity score is lost with the multidesign option.

As the results can highly fluctuate according to the informativity and the density of available markers, it is possible to perform a simulation to define the best suited window sizes percentage. It is also possible to combine this simulation with all available options (resampling and multidesign).

In order to test the program core functions and options, MarkerSet has been run on several different data files. First, a small data file corresponding to a real case has been generated with 206 low informativity markers (among them, 162 are not informative at all), located on one chromosome of 63 Mb and 4 tested animals. Using MarkerSet with this file in verbose mode, we have checked that the algorithm selects effectively the best informative marker taking into consideration the marker location in case of similar informativity, but also enlarges the window in case of low or no informativity. The resampling function has also been validated. The test file and the results log are available on the website as examples.

**Figure 4:**
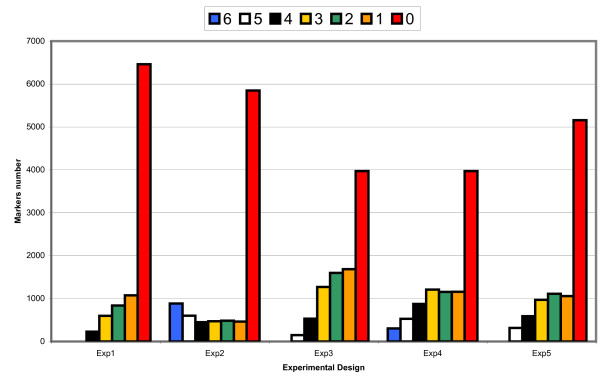
Experimental designs marker informativity distribution. Each bar represents the number of markers for every informativity values for each experimental design.

Once the main concept of the program was tested and validated with the small data file, we have extended the functioning of the program to other various situations by generating simulated data files with different marker density and informativity distribution. Finally, the program has been also tested on a real informativity file of 9216 markers with five experimental designs (cf. Figure 4 for the distribution of the number of maker in each informativity value). For each informativity file, a selection of 384 markers has been performed in the basic mode with or without resampling option, and in the multidesign mode (1536 markers requested) with or without resampling option. Score results for simulated data files and real data file are shown in table 1 and 2, respectively (see additional files 1 and 2 for complete results). As expected, MarkerSet results are very sensitive to marker density and informativity distribution. It is noticeable that, with our real data file, there are not enough informative markers to select 1536 SNP. Moreover, multidesign option could have a drastic impact on the scores and the loss of informativity (Ratio) with low informativity files (especially with the resampling option).

**Table 1:** Testing results for simulated data files, requesting 384 and 1536 markers.

		**Low Density (5K)**			**High Density (40K)**		
		Score	Ratio	-r gain	Score	Ratio	-r gain

Basic	HI	1002.86			1051.61		
	VI	927.44			1047.9		
	LI	317.92			500		
R	HI	1008.3		0.54%	1051.61		0%
	VI	932.38		0.53%	1047.9		0%
	LI	320.59		0.84%	500		0%
MD	MD	1879.48			3513.19		
	HI	2774.49	0.99		3959.82	0.96	
	VI	1940.27	0.96		3760.68	0.94	
	LI	428.14	0.80		769.18	0.49	
R + MD	MD	2428.19		29.19%	3513.19		0%
	HI	3487.45	0.98	25.70%	3959.82	0.96	0%
	VI	2519.01	0.82	29.83%	3760.68	0.94	0%
	LI	539.56	0.53	26.02%	769.18	0.49	0%

For this purpose, six files with different marker informativity status have been generated with two variables. The first one is the marker density (5K markers – LD for low density or 40K markers – HD for High Density spanned homogeneously on the genome). The second one is marker informativity distribution. Considering a total of 100 reference animals, the following conditions have been explored: markers with heterozygozity values ranging from 50 to 100 (High Informativity, HI), 0 to 100 (Various Informativity, VI) or 0 to 50 (Low Informativity, LI). For each markers panel and condition, the maximal available informativity score (max info), the selected set score, the multidesign/monodesign ratio and the score gain obtained by using the resampling options (-r gain) are detailed. R and MD refer at resampling option activation and multidesign option activation respectively. Scores results are depending on marker density and informativity distribution (better with HI and lower with LI files). Nevertheless, there's only a slight score difference between HI and VI, showing the efficiency of MarkerSet to select the most informative markers. Resampling option is more useful with LD files but can have an impact on the loss of informativity (Ratio) in multidesign mode with LI file.

**Table 2:** Testing results for the real data set, requesting 384 and 1536 markers.

		**Real dataset**									
		Max info	Score	Ratio	-r gain	Dmax	Dmin	AveD	StD	Markers	0 markers

Basic	Exp1	3509.86	528.67			28.6	0.4	9.2	2.2	380	72
	Exp2	5958.31	808.76			28.7	0.4	9.2	2.2	380	32
	Exp3	5685.11	680.55			20.6	0.6	9.2	2	380	10
	Exp4	6503.60	785.52			17.8	0.2	9.1	2.1	382	7
	Exp5	5293.64	673.66			17.5	0.2	9.1	2	382	26

R	Exp1		605.03		14.44%	90.3	0.4	9.5	5.8	366	0
	Exp2		887.82		9.78%	20.6	0.3	9.2	2.5	383	0
	Exp3		701.85		3.13%	16.8	0.5	9.1	2.1	384	0
	Exp4		803.9		2.34%	16.6	0	9.1	2.1	384	0
	Exp5		713.43		5.90%	26.9	0.5	9.2	2.5	380	0

MD	MD	3581.64	979.58			11.7	0.1	2.5	0.9	1461	137
	Exp1		801.79	0.81							
	Exp2		1512.85	0.92							
	Exp3		1282.7	0.89							
	Exp4		1494.43	0.9							
	Exp5		1229.34	0.89							

R + MD	MD		1114.33		13.76%	11.3	0.1	2.4	0.9	1483	0
	Exp1		898.48	0.47	12.06%						
	Exp2		1720.82	0.6	13.75%						
	Exp3		1446.47	0.72	12.77%						
	Exp4		1695.42	0.73	13.45%						
	Exp5		1400.07	0.63	13.89%						

The data file includes the genotype of 9216 SNPs covering the whole genome for The 26 F1 sires of five real chicken F2 designs (4 in Exp1, 5 in Exp3 and Exp5 and 6 in Exp2 and Exp4). For each markers panel and condition, the maximal available informativity score (max info), the selected set score, the multidesign/monodesign ratio, the score gain obtained by using the resampling (-r gain), the maximal (Dmax), minimal (Dmin), average (AveD) and standard deviation (StD) distances between two markers, the number of selected markers and the number of no informative markers in this set are detailed. R and MD refer at resampling option activation and multidesign option activation, respectively. With the resampling option, the gain is inversely proportional to the maximum informativity, except for Exp2, because of an overrepresentation of markers heterozygous for 0 and 6 animals in this experimental design. The results for multidesign mode (1536 markers) are similar to those obtained with the 5K markers file: the ratio is about 0.90, and the resampling option permits the increase of the number of selected markers (and thus the final score) without significant modifications of the average distance and the standard deviation.

**Figure 5:**
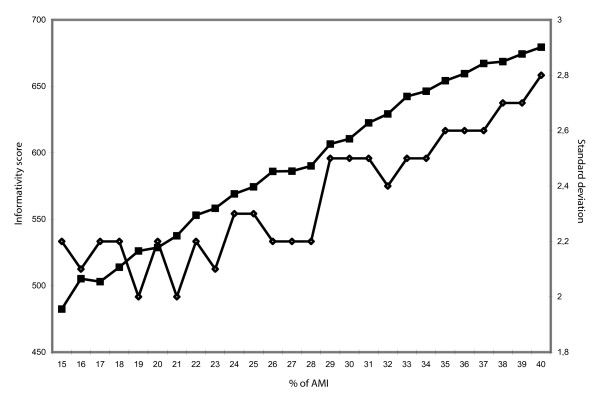
Impacts of window sizes upon informativity score and standard deviation The horizontal axis represents the percentage of AMI used to define the window size (15 to 40%). The left vertical axis represents the best marker set score (full squares), and the right vertical axis the standard deviation (white diamonds). The simulation mode was performed on experimental design 1 for 384 markers requested without the resampling options.

The simulation option has been also tested for simulated data and real data files (see additional files 1 and 2). As expected, the highest score is always obtained with the highest AMI percentage since the window sizes are larger (see Figure 5). Depending on the priority given to the marker locations or their informativity, users should test different conditions to find out which parameters are best fitted to their experimental designs.

## Availability and requirements

Project name: MarkerSet

Project homepage: 

Operating system: Platform independent

Programming language: PERL

Other requirements: POSIX PERL module

License: GNU GPL

Any restrictions to use by non-academics: license needed

## Competing interests

The authors declare that they have no competing interests.

## Authors' contributions

OD carried out the general objectives of the program, data analyses and drafts the manuscript. FL carried out the general objectives of the program, algorithms definition, PERL code implementation and drafts the manuscript. Both authors read and approved the final manuscript.

## Supplementary Material

Additional file 1 complete results for 5K and 40K markers datasets (including simulation mode results). Click here for file

Additional file 2 Complete results for real dataset (including simulation mode results). Click here for file

## References

[B1] Lander ES, Linton LM, Birren B, Nusbaum C, Zody MC, Baldwin J, Devon K, Dewar K, Doyle M, FitzHugh W, Funke R, Gage D, Harris K, Heaford A, Howland J, Kann L, Lehoczky J, LeVine R, McEwan P, McKernan K, Meldrim J, Mesirov JP, Miranda C, Morris W, Naylor J, Raymond C, Rosetti M, Santos R, Sheridan A, Sougnez C, Stange-Thomann N, Stojanovic N, Subramanian A, Wyman D, Rogers J, Sulston J, Ainscough R, Beck S, Bentley D, Burton J, Clee C, Carter N, Coulson A, Deadman R, Deloukas P, Dunham A, Dunham I, Durbin R, French L, Grafham D, Gregory S, Hubbard T, Humphray S, Hunt A, Jones M, Lloyd C, McMurray A, Matthews L, Mercer S, Milne S, Mullikin JC, Mungall A, Plumb R, Ross M, Shownkeen R, Sims S, Waterston RH, Wilson RK, Hillier LW, McPherson JD, Marra MA, Mardis ER, Fulton LA, Chinwalla AT, Pepin KH, Gish WR, Chissoe SL, Wendl MC, Delehaunty KD, Miner TL, Delehaunty A, Kramer JB, Cook LL, Fulton RS, Johnson DL, Minx PJ, Clifton SW, Hawkins T, Branscomb E, Predki P, Richardson P, Wenning S, Slezak T, Doggett N, Cheng JF, Olsen A, Lucas S, Elkin C, Uberbacher E, Frazier M, Gibbs RA, Muzny DM, Scherer SE, Bouck JB, Sodergren EJ, Worley KC, Rives CM, Gorrell JH, Metzker ML, Naylor SL, Kucherlapati RS, Nelson DL, Weinstock GM, Sakaki Y, Fujiyama A, Hattori M, Yada T, Toyoda A, Itoh T, Kawagoe C, Watanabe H, Totoki Y, Taylor T, Weissenbach J, Heilig R, Saurin W, Artiguenave F, Brottier P, Bruls T, Pelletier E, Robert C, Wincker P, Smith DR, Doucette-Stamm L, Rubenfield M, Weinstock K, Lee HM, Dubois J, Rosenthal A, Platzer M, Nyakatura G, Taudien S, Rump A, Yang H, Yu J, Wang J, Huang G, Gu J, Hood L, Rowen L, Madan A, Qin S, Davis RW, Federspiel NA, Abola AP, Proctor MJ, Myers RM, Schmutz J, Dickson M, Grimwood J, Cox DR, Olson MV, Kaul R, Shimizu N, Kawasaki K, Minoshima S, Evans GA, Athanasiou M, Schultz R, Roe BA, Chen F, Pan H, Ramser J, Lehrach H, Reinhardt R, McCombie WR, de la Bastide M, Dedhia N, Blocker H, Hornischer K, Nordsiek G, Agarwala R, Aravind L, Bailey JA, Bateman A, Batzoglou S, Birney E, Bork P, Brown DG, Burge CB, Cerutti L, Chen HC, Church D, Clamp M, Copley RR, Doerks T, Eddy SR, Eichler EE, Furey TS, Galagan J, Gilbert JG, Harmon C, Hayashizaki Y, Haussler D, Hermjakob H, Hokamp K, Jang W, Johnson LS, Jones TA, Kasif S, Kaspryzk A, Kennedy S, Kent WJ, Kitts P, Koonin EV, Korf I, Kulp D, Lancet D, Lowe TM, McLysaght A, Mikkelsen T, Moran JV, Mulder N, Pollara VJ, Ponting CP, Schuler G, Schultz J, Slater G, Smit AF, Stupka E, Szustakowski J, Thierry-Mieg D, Thierry-Mieg J, Wagner L, Wallis J, Wheeler R, Williams A, Wolf YI, Wolfe KH, Yang SP, Yeh RF, Collins F, Guyer MS, Peterson J, Felsenfeld A, Wetterstrand KA, Patrinos A, Morgan MJ, Szustakowki J, de Jong P, Catanese JJ, Osoegawa K, Shizuya H, Choi S, Chen YJ (2001). Initial sequencing and analysis of the human genome. Nature.

[B2] Wong GK, Liu B, Wang J, Zhang Y, Yang X, Zhang Z, Meng Q, Zhou J, Li D, Zhang J, Ni P, Li S, Ran L, Li H, Li R, Zheng H, Lin W, Li G, Wang X, Zhao W, Li J, Ye C, Dai M, Ruan J, Zhou Y, Li Y, He X, Huang X, Tong W, Chen J, Ye J, Chen C, Wei N, Dong L, Lan F, Sun Y, Yang Z, Yu Y, Huang Y, He D, Xi Y, Wei D, Qi Q, Li W, Shi J, Wang M, Xie F, Zhang X, Wang P, Zhao Y, Li N, Yang N, Dong W, Hu S, Zeng C, Zheng W, Hao B, Hillier LW, Yang SP, Warren WC, Wilson RK, Brandstrom M, Ellegren H, Crooijmans RP, van der Poel JJ, Bovenhuis H, Groenen MA, Ovcharenko I, Gordon L, Stubbs L, Lucas S, Glavina T, Aerts A, Kaiser P, Rothwell L, Young JR, Rogers S, Walker BA, van Hateren A, Kaufman J, Bumstead N, Lamont SJ, Zhou H, Hocking PM, Morrice D, de Koning DJ, Law A, Bartley N, Burt DW, Hunt H, Cheng HH, Gunnarsson U, Wahlberg P, Andersson L, Kindlund E, Tammi MT, Andersson B, Webber C, Ponting CP, Overton IM, Boardman PE, Tang H, Hubbard SJ, Wilson SA, Yu J, Yang H (2004). A genetic variation map for chicken with 2.8 million single-nucleotide polymorphisms. Nature.

[B3] Burkett KM, Ghadessi M, McNeney B, Graham J, Daley D (2005). A comparison of five methods for selecting tagging single-nucleotide polymorphisms. BMC Genet.

[B4] Carlson CS, Eberle MA, Rieder MJ, Yi Q, Kruglyak L, Nickerson DA (2004). Selecting a maximally informative set of single-nucleotide polymorphisms for association analyses using linkage disequilibrium. Am J Hum Genet.

[B5] He J, Zelikovsky A (2004). Linear reduction methods for tag SNP selection. Conf Proc IEEE Eng Med Biol Soc.

[B6] He J, Zelikovsky A (2007). Informative SNP selection methods based on SNP prediction. IEEE Trans Nanobioscience.

[B7] Nicolas P, Sun F, Li LM (2006). A model-based approach to selection of tag SNPs. BMC Bioinformatics.

[B8] Qin ZS, Gopalakrishnan S, Abecasis GR (2006). An efficient comprehensive search algorithm for tagSNP selection using linkage disequilibrium criteria. Bioinformatics.

[B9] Sham PC, Ao SI, Kwan JS, Kao P, Cheung F, Fong PY, Ng MK (2007). Combining functional and linkage disequilibrium information in the selection of tag SNPs. Bioinformatics.

[B10] Stram DO (2004). Tag SNP selection for association studies. Genet Epidemiol.

[B11] Stram DO (2005). Software for tag single nucleotide polymorphism selection. Hum Genomics.

